# Changes Consequent on the Death of the Queen

**Published:** 1901-02-09

**Authors:** 


### INSTITUTIONAL QUESTIONS.

### CHANGES CONSEQUENT ON THE DEATH OF THE QUEEN.

"Hospital Secretary" writes: Some authoritative information in your next issue in reference to changes consequent on the death of the Queen would be welcomed by many connected with institutions under Royal Patronage. The points on which difference of opinion exists seem to be:—(1) The position of institutions of which our King and Queen were Patrons or Presidents as Prince and Princess of Wales. Does such patronage lapse *ipso facto* on accession? If so, how, when, and through whom, should steps be taken for its renewal. (2) To whom, and when, should resolutions of condolence be forwarded? (3) Should mourning letter-paper be used by such institutions?

[(1) Strictly speaking the patronage lapses. In such circumstances it is customary in preparing the address of condolence and congratulation to insert a clause expressing a hope that His Majesty or Their Majesties will continue the Royal patronage or become the President or Presidents of the institution. (2) The Home Secretary. (3) In the case of those institutions where the Queen was patron, it is usual for mourning paper to be used.—Ed. The Hospital.]

